# Vagus nerve stimulation for pharmacoresistant epilepsy secondary to encephalomalacia: A single-center retrospective study

**DOI:** 10.3389/fneur.2022.1074997

**Published:** 2023-01-06

**Authors:** Mengyi Guo, Jing Wang, Zhonghua Xiong, Jiahui Deng, Jing Zhang, Chongyang Tang, Xiangru Kong, Xiongfei Wang, Yuguang Guan, Jian Zhou, Feng Zhai, Guoming Luan, Tianfu Li

**Affiliations:** ^1^Beijing Key Laboratory of Epilepsy Research, Department of Brain Institute, Center of Epilepsy, Beijing Institute for Brain Disorders, Sanbo Brain Hospital, Capital Medical University, Beijing, China; ^2^Department of Neurology, Center of Epilepsy, Beijing Institute for Brain Disorders, Sanbo Brain Hospital, Capital Medical University, Beijing, China; ^3^Beijing Key Laboratory of Epilepsy Research, Department of Neurosurgery, Center of Epilepsy, Beijing Institute for Brain Disorders, Sanbo Brain Hospital, Capital Medical University, Beijing, China

**Keywords:** encephalomalacia, pharmacoresistant epilepsy, vagus nerve stimulation, effectiveness, predictor

## Abstract

**Objective:**

Vagus nerve stimulation (VNS) is an adjunctive treatment for pharmacoresistant epilepsy. Encephalomalacia is one of the most common MRI findings in the preoperative evaluation of patients with pharmacoresistant epilepsy. This is the first study that aimed to determine the effectiveness of VNS for pharmacoresistant epilepsy secondary to encephalomalacia and evaluate the potential predictors of VNS effectiveness.

**Methods:**

We retrospectively analyzed the seizure outcomes of VNS with at least 1 year of follow-up in all patients with pharmacoresistant epilepsy secondary to encephalomalacia. Based on the effectiveness of VNS (≥50% or <50% reduction in seizure frequency), patients were divided into two subgroups: responders and non-responders. Preoperative data were analyzed to screen for potential predictors of VNS effectiveness.

**Results:**

A total of 93 patients with epilepsy secondary to encephalomalacia who underwent VNS therapy were recruited. Responders were found in 64.5% of patients, and 16.1% of patients achieved seizure freedom at the last follow-up. In addition, the responder rate increased over time, with 36.6, 50.5, 64.5, and 65.4% at the 3-, 6-, 12-, and 24-month follow-ups, respectively. After multivariate analysis, seizure onset in adults (>18 years old) (OR: 0.236, 95%CI: 0.059–0.949) was found to be a positive predictor, and the bilateral interictal epileptic discharges (IEDs) (OR: 3.397, 95%CI: 1.148–10.054) and the bilateral encephalomalacia on MRI (OR: 3.193, 95%CI: 1.217–8.381) were found to be negative predictors of VNS effectiveness.

**Conclusion:**

The results demonstrated the effectiveness and safety of VNS therapy in patients with pharmacoresistant epilepsy secondary to encephalomalacia. Patients with seizure onset in adults (>18 years old), unilateral IEDs, or unilateral encephalomalacia on MRI were found to have better seizure outcomes after VNS therapy.

## 1. Introduction

Focal encephalomalacia is a common structural brain lesion detected during magnetic resonance imaging (MRI) in patients with pharmacoresistant epilepsy (1, 2). The etiology of encephalomalacia includes brain trauma, perinatal hypoxia, infection, intracranial hematoma, surgical procedures, as well as some unknown factors (3). Although the encephalomalacia alone may not cause seizures, the surrounding scars may interfere with the normal electrophysiological activity of neurons and cause hyperplastic glial dysfunction, which in turn leads to abnormal discharge associated with seizures (4, 5). Patients with pharmacoresistant epilepsy secondary to encephalomalacia are usually resistant to anti-seizure medications, and surgical intervention is another widely accepted treatment option (3, 6). However, in conditions of widely distributed encephalomalacia involved in eloquent brain regions or bilateral hemispheres, patients are not good candidates for resection (5, 7). Thus, for those unsuitable for surgical therapy or with unsatisfactory surgical outcomes, it is urgent to explore novel therapeutic strategies.

Since its first reported use in humans in 1988 and more than 100,000 subsequent implantations, vagus nerve stimulation (VNS) has become a reliable method of treating patients with pharmacoresistant epilepsy who are not good candidates for epilepsy surgery or in whom surgery resulted in no benefit (8). According to the results of randomized controlled trials (9), meta-analyses (10), and retrospective studies (11, 12), ~50–60% of patients achieve a seizure reduction of ≥50% after VNS surgery, with a rate of complete seizure freedom ranging from 6 to 8%. Predictors of VNS effectiveness are a focus of related research at present. Several potential predictors updated recently include brain connectomic profiling (13), heart rate variability (14), and genetic variations of adenosine kinase (15). The effectiveness and safety of VNS are also demonstrated in some specific types of epilepsy, such as tuberous sclerosis complex (16), Lennox–Gastaut syndrome (17), post-encephalitic epilepsy (18), and post-traumatic epilepsy (19). Based on the advantages of VNS therapy, it may shed some light on the therapy of pharmacoresistant epilepsy secondary to encephalomalacia.

Various causes can lead to encephalomalacia in the brain, such as stroke (20, 21), head trauma (19, 22, 23), and encephalitis (18, 24), in which the effectiveness of VNS for epilepsy has been reported, separately. This present study aimed to demonstrate the effectiveness of VNS in 93 patients with pharmacoresistant epilepsy secondary to encephalomalacia under different conditions, as well as to evaluate potential predictors for VNS effectiveness.

## 2. Materials and methods

### 2.1. Patients

**Figure 1:**
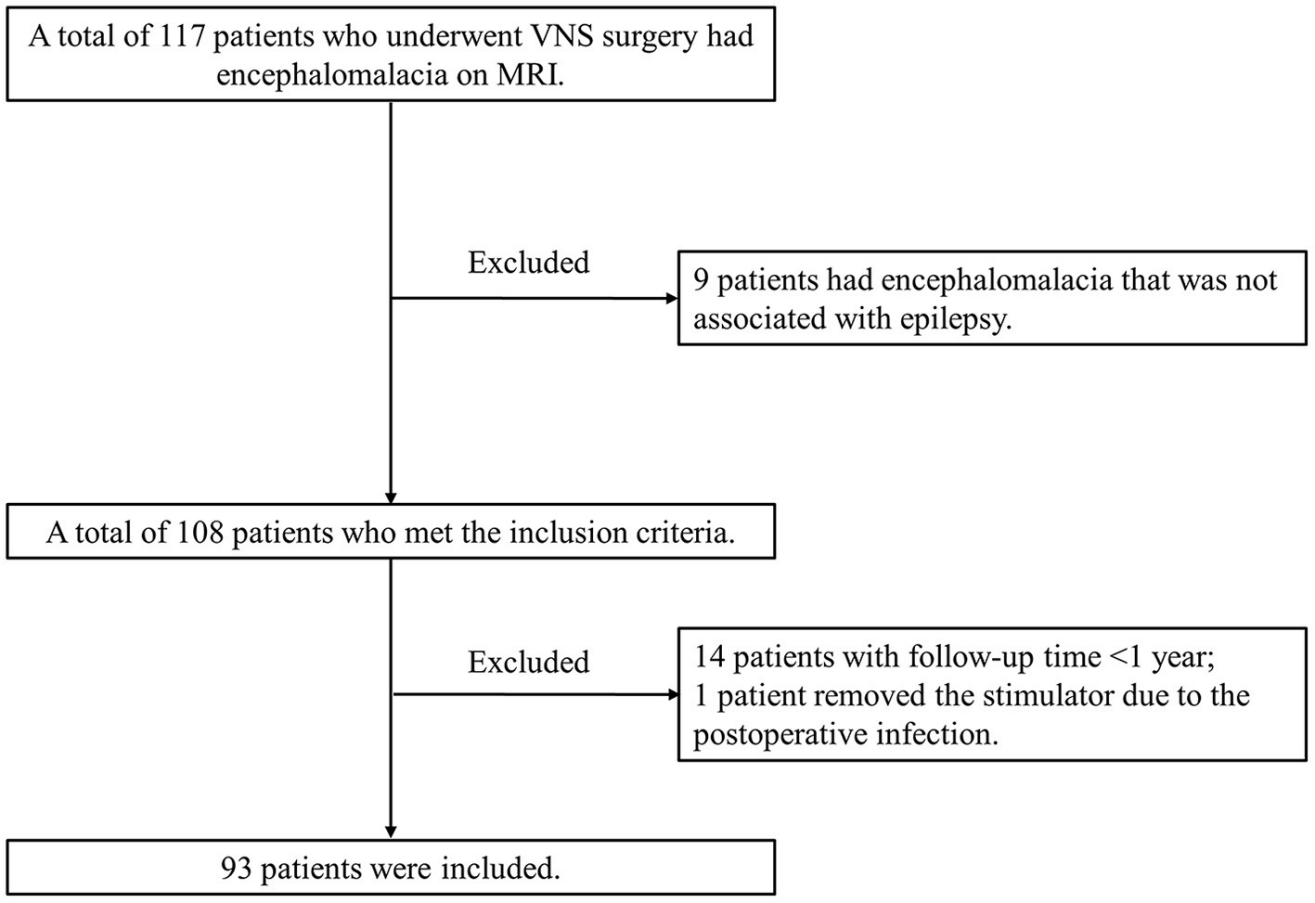
Flowchart for recruiting patients who satisfy the inclusion and exclusion criteria.

**Figure 2:**
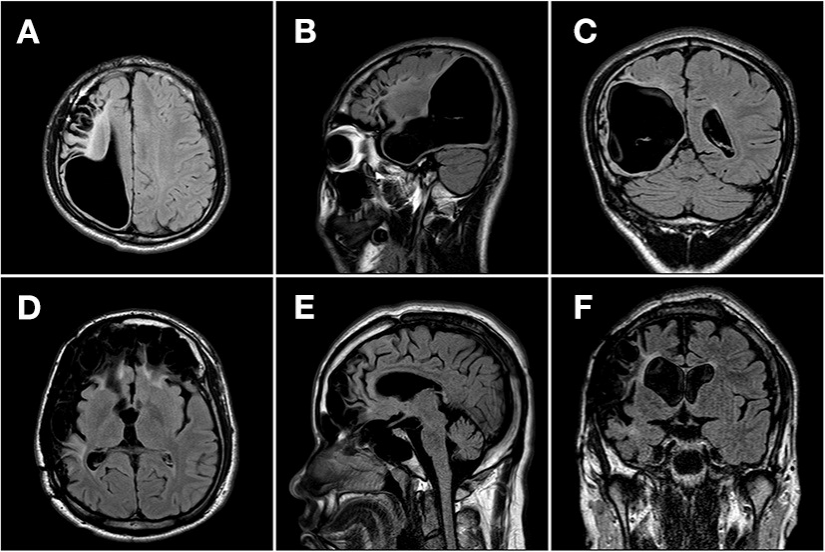
Representative MR images of patients with pharmacoresistant epilepsy secondary to encephalomalacia. Representative MR images (T2-weighted fluid-attenuated inversion recovery image) of two patients with pharmacoresistant epilepsy secondary to encephalomalacia in the axial **(A, D)**, sagittal **(B, E)**, and coronal **(C, F)** planes. **(A–C)** A 17-year-old boy with unilateral encephalomalacia on MRI due to intracranial hematoma. The encephalomalacia was observed in the right frontal, parietal, and temporal lobes. The patient got a reduction of 80% in seizure frequency after 1 year following the VNS therapy. **(D–F)** A 24-year-old man with bilateral encephalomalacia on MRI due to head trauma. The encephalomalacia was observed in the right temporal and parietal lobes, as well as in bilateral frontal lobes. The patient got no reduction in seizure frequency during a 3-year follow-up after the VNS therapy.

We retrospectively studied VNS effectiveness in patients with pharmacoresistant epilepsy who received VNS implantation from Sanbo Brain Hospital, Capital Medical University, between September 2008 and April 2021. All enrolled patients had evidence of encephalomalacia on brain MRI. Encephalomalacia in this study was defined as a loss of parenchymal thickness accompanied by laminar necrosis in the brain (5, 25, 26). Representative MR images of encephalomalacia are shown in **Figure 2**. The inclusion criteria for enrolled patients were as follows: (1) patients with pharmacoresistant epilepsy who received VNS therapy; (2) patients with evidence of encephalomalacia on brain MRI; and (3) patients whose MRI findings of encephalomalacia were associated with epilepsy after detailed preoperative evaluation. Thus, those with pharmacoresistant epilepsy secondary to encephalomalacia who received VNS therapy were included in this study (Figure 1). All recruited patients were followed up by at least 1 year. Detailed demographic and clinical information were collected from the medical records.

This study complied with the World Medical Association Declaration of Helsinki published on the website of the Journal of American Medical Association and was approved by the Ethics Committee of Sanbo Brain Hospital, Capital Medical University (SBNK-2017-15-01). Written informed consent was obtained from all patients or their guardians.

### 2.2. Preoperative evaluation

Patients in our comprehensive epilepsy center were all evaluated by MRI and video electroencephalography (VEEG) before the operation. Some patients further received positron emission tomography-computed tomography (PET-CT), magnetoencephalography (MEG), and neuropsychological assessments. At a multidisciplinary team (MDT) conference, all results of the preoperative evaluation were analyzed in detail by experienced neurologists, neurosurgeons, neuroradiologists, and electrophysiologists, to determine treatment strategies for each patient (16). Based on our previous strategies (16), VNS was recommended in the following conditions: (1) patients whose epileptogenic focus could not be accurately localized; (2) patients with epileptogenic focus overlapping with the eloquent areas, which could be determined by SEEG and the Wada test; (3) patients who did not accept surgical resection; and (4) patients with early surgical failure. VNS implantations were conducted by two neurosurgeons according to standard procedures (27). Based on available guidelines (28), the stimulation parameters were adjusted routinely after device implantation.

### 2.3. Programming strategy of VNS

The parameter setting of VNS was conducted based on our previous programming strategy (16). In the 93 patients recruited in our study, two models of vagus nerve stimulators were implanted: Model 103 (Demipulse, LivaNova, England) implanted in 61.3% (57/93) of patients, and Model G111 (Beijing PINS Medical Co., Ltd, China) implanted in 38.7% (36/93) of patients. After 7 days of the stimulator implantation, the stimulation was initiated. For the initial parameter settings, the out current was set as 0.5 mA, the signal on time was set as 30 s, and the signal off time was set as 5 min. The signal frequency (30 Hz) and the pulse width (250 μs) were kept consistent, and the magnet current was set as 0.25 mA higher than the output current. The out current was elevated to 1.25–1.5 mA in 1 month at the outpatient clinic. From then on, the parameters would be modified to 0.25 mA every 3–6 months based on improvements in seizure control and tolerance of patients.

### 2.4. Clinical data collection

The collected medical history of patients included sex, age of VNS implantation, age of epilepsy onset, epilepsy duration, predominant type and frequency of seizures, number of preoperative anti-seizure medications (ASMs), preoperative neurological deficit, history of status epilepticus (SE), the spatial distribution of EEG, and encephalomalacia on brain MRI. Detailed information on antecedent events of encephalomalacia was analyzed in patients with specific etiology of encephalomalacia, including the type of etiology, age of etiology, and the interval between etiology and the first seizure.

According to the medical documents, the seizure type of each patient was defined as the most frequent seizure type, which was classified as “focal onset” and “generalized onset” based on the 2017 ILAE classification of epilepsy (29). The duration of follow-up was divided into “ ≤2,” “2–6,” and “≥6” years.

### 2.5. MRI

Brain 1.5-T MRI scans were conducted in all included patients, including T1-weighted, T2-weighted, and T2-weighted fluid-attenuated inversion recovery (FLAIR) sequences. Encephalomalacia was defined as a loss of parenchymal thickness accompanied by laminar necrosis in the brain (5, 25, 26). The MR images of all patients were reviewed and classified as follows: (1) unilateral: the encephalomalacia showed by MRI involved only one hemisphere; and (2) bilateral: the encephalomalacia showed by MRI involved both hemispheres. Based on our previous study, the image archiving and communication system of Hinacom Software and Technology was used to define the regions of the responsible lesion (3). The lesions were inspected by a group of experienced neuroradiologists, neurologists, and neurosurgeons.

### 2.6. Scalp EEG findings

All patients were monitored with 64-channel long-term video EEG for at least 24 h using a standard 10–20 electrode placement system. The interictal epileptic discharges (IEDs) were divided into two types: (1) unilateral: the IEDs involved only one hemisphere; or (2) bilateral: the IEDs involved both hemispheres and were either diffused or generalized. Similarly, for patients whose seizures were recorded, the ictal onset rhythms were also classified as unilateral or bilateral. Of note, concordance of the interictal and ictal EEG findings was defined as localization of the interictal and ictal epileptic discharges in the same brain region or hemisphere.

### 2.7. Magnetoencephalography

A total of 36 (38.7%) patients underwent MEG. Concordance of the IEDs and MEG findings was defined as the localization of the IEDs and MEG spike sources to the same brain region.

### 2.8. Seizure outcome and follow-up

All enrolled patients were followed for at least 1 year after VNS therapy. The seizure outcomes were collected by questionnaire when patients were readmitted for adjustment of stimulation parameters or online remote follow-up. Based on our previous study (18), patients with a reduction of over 50% in baseline seizure frequency of the predominant seizure type were defined as responders. Seizure freedom in this study referred to the complete freedom of all types of seizures at the last follow-up. The seizure outcomes were collected at 3, 6, 12, and 24 months and the last follow-up after VNS surgery. Results at the last follow-up were used to define the overall effectiveness and potential predictors of VNS.

### 2.9. Statistical analysis

The SPSS Software version 23.0 was used for all analyses. All calculated *P*-values in the present study were two-tailed, and a *p*-value of <0.05 was considered statistically significant. Categorical variables were shown as frequencies. Pearson's chi-square or Fisher's exact test was used for univariate analysis. To determine the threshold of continuous variables that may predict seizure outcomes, continuous variables were stratified using a receiver operating curve analysis, and the cutoff values were determined according to Youden's index. Variables showing a *p*-value <0.05 in the univariate analysis were then entered into a multivariate logistic regression model in a backward manner.

## 3. Results

### 3.1. Demographic characteristics

The overall process of patient enrollment is shown in Figure 1. Of the 108 patients with pharmacoresistant epilepsy secondary to encephalomalacia who met the inclusion criteria, 14 patients with a follow-up of <1 year and 1 patient removed the stimulator due to post-operative infection. This study was based on the remaining 93 patients (77 men and 16 women) managed during 2008–2021. The most frequently reported adverse events included voice hoarse, coughing, and throat pain, while all the side effects above were tolerable and transient.

Among all included patients, the median (interquartile range, IQR) age of VNS implantation, age at seizure onset, and duration of seizures were 20.0 (IQR 13.4–29.5) years, 9.0 (IQR 5.0–17.0) years, and 6.0 (IQR 2.6–14.6) years, respectively. Notably, 11 (11.8%) patients had a history of SE, and aura occurred in 22 (23.7%) patients at the beginning of seizures. There were 29 (31.2%) patients accompanied by preoperative neurological deficits: 23 (24.6%) reported hemiparesis, 1 (1.1%) reported aphasia, 2 (2.2%) reported both hemiparesis and aphasia, 2 (2.2%) reported ataxia, and 1 (1.1%) was defined as a persistent vegetative state. Based on the medical records, antecedent events of encephalomalacia were found in 78 (83.9%) patients: 34 (36.6%) had head trauma, 17 (18.3%) had perinatal hypoxia, 17 (18.3%) had meningoencephalitis, 3 (3.2%) had undergone previous surgical procedures, and 7 (7.5%) had an intracranial hematoma. The median age of the antecedent events was 5.0 (IQR: 0.0–17.0) years, and the median interval between the antecedent events and the first seizure was 2.0 (IQR: 0.1–6.0) years. Other patient characteristics are shown in Table 1. Besides, we also evaluated the comparison of demographic characteristics between patients who got seizure freedom at the last follow-up and the others. Detailed information is shown in Supplementary Table 1.

**Table 1:** Patients' demographic and clinical features.

**Variables**	**Total (*n* = 93)**	**Responder (*n* = 60)**	**Non-responder (*n* = 33)**	***P*-value**
Male, *n* (%)	77 (82.8)	52 (86.7)	25 (75.8)	0.148
Age at VNS implantation, year old				0.126
≤ 12	20 (21.5)	10 (16.7)	10 (30.3)	
>12	73 (78.5)	50 (83.3)	23 (69.7)	
Age at seizure onset, year old				0.021^*^
≤ 18	72 (77.4)	42 (70.0)	30 (90.9)	
>18	21 (22.6)	18 (30.0)	3 (9.1)	
Duration of epilepsy, year				0.032^*^
≤ 15	71 (76.3)	50 (83.3)	21 (63.6)	
>15	22 (23.7)	10(16.7)	12 (36.4)	
Seizure type, *n* (%)				0.597
Focal onset	82 (88.2)	53 (88.3)	29 (87.9)	
Generalized onset	11 (11.8)	7 (11.7)	4 (12.1)	
Monthly seizure frequency				0.911
≤ 5	43 (46.2)	28(46.7)	15 (45.5)	
>5	50 (53.8)	32 (53.3)	18 (54.5)	
Aura, *n* (%)				0.921
Yes	22 (23.7)	14 (23.3)	8 (24.2)	
No	71 (76.3)	46 (76.7)	25 (75.8)	
Types of ASMs				0.358
≤ 2	62 (66.7)	42 (70.0)	20 (60.6)	
>2	31 (33.3)	18 (30.0)	13 (39.4)	
Etiology				0.900
Head trauma	34 (36.6)	22 (36.7)	12 (36.5)	
Perinatal hypoxia	17 (18.3)	10 (16.7)	7 (21.2)	
Meningoencephalitis	17 (18.3)	13 (21.7)	4 (12.1)	
Previous surgical procedure	3 (3.2)	2 (3.3)	1 (3.0)	
Intracranial hematoma	7 (7.5)	4 (6.6)	3 (9.1)	
Unknown	15 (16.1)	9 (15.0)	6 (18.1)	
Age of etiology, year old				0.077
≤ 20	61 (65.6)	36 (60.0)	25 (75.7)	
>20	17 (18.3)	15 (25.0)	2 (6.1)	
Unknown	15 (16.1)	9 (15.0)	6 (18.2)	
Interval between etiology and the first seizure, year				0.202
≤ 8	68 (73.1)	42 (70.0)	26 (78.8)	
>8	10 (10.8)	9 (15.0)	1 (3.0)	
Unknown	15 (16.1)	9 (15.0)	6 (18.2)	
Preop neurological deficit, *n* (%)	29 (31.2)	16 (26.7)	13 (39.4)	0.205
History of SE, *n* (%)	11 (11.8)	8 (13.3)	3 (9.1)	0.741
Spatial distribution of IEDs, *n* (%)				0.010^*^
Unilateral	33 (35.5)	27 (45.0)	6 (18.2)	
Bilateral	60 (64.5)	33 (55.0)	27 (81.8)	
Ictal onset rhythms of EEG, *n* (%)				0.777
Unilateral	17 (18.3)	10 (16.7)	7 (21.2)	
Bilateral	56 (60.2)	36 (60.0)	20 (60.6)	
Unknown	20 (21.5)	14 (23.3)	6 (18.2)	
Concordance of IEDs and ictal onset rhythms				0.510
Yes	46 (49.5)	27 (45.0)	19 (57.6)	
No	27 (29.0)	19 (31.7)	8 (24.2)	
Unknown	20 (21.5)	14 (23.3)	6 (18.2)	
Encephalomalacia on MRI				0.045^*^
Unilateral	44 (47.3)	33 (55.0)	11 (33.3)	
Bilateral	49 (52.7)	27 (45.0)	22 (66.7)	
Site of encephalomalacia				0.444
Frontal lobe	10 (10.8)	6 (10.0)	4 (12.1)	
Temporal lobe	6 (6.5)	5 (8.3)	1 (3.0)	
Parietal lobe	4 (4.3)	4 (6.7)	0 (0.0)	
Occipital lobe	4 (4.3)	2 (3.3)	2 (6.1)	
Multilobar	69 (74.1)	43 (71.7)	26 (78.8)	
Performance of MEG, *n* (%)				0.480
Yes	36 (38.7)	21 (35.0)	15 (45.5)	
No	57 (61.3)	39 (65.0)	18 (54.5)	
Concordance of MEG and IEDs				0.573
Yes	23 (24.7)	13 (21.7)	10 (30.3)	
No	10 (10.8)	6 (10.0)	4 (12.1)	
Unknown^a^	60 (64.5)	41 (68.3)	19 (57.6)	
The type of stimulator				0.377
Model 103	57 (61.3)	39 (65.0)	18 (55.5)	
Model G111	36 (38.7)	21 (35.0)	15 (45.5)	
Time of the last follow-up, year				0.673
≤ 2	28 (30.1)	18 (30.0)	10 (30.3)	
2–6	56 (60.2)	35 (58.3)	21 (63.6)	
≥6	9 (9.7)	7 (11.7)	2 (6.1)	

ASMs, anti-seizure medications; EEG, electroencephalogram; IEDs, interictal epileptiform discharges; MEG, magnetoencephalography; MRI, magnetic resonance imaging; VNS, vagus nerve stimulation; SE, status epilepticus;

* *P* < 0.05;

a MEG was performed in three of these patients, but the spikes sources were not detected.

### 3.2. MRI results

Brain MRI results were reviewed in all patients. Encephalomalacia was observed in only one hemisphere in 44 (47.3%) patients, and in the other 49 (52.7%) patients, encephalomalacia was found in both hemispheres. Representative MR images are shown in Figure 2. Among the 93 patients in this study, encephalomalacia in 10 (10.8%) patients involved the frontal lobe, 6 (6.5%) patients involved the temporal lobe, 4 (4.3%) patients involved the parietal lobe, 4 (4.3%) patients involved the occipital lobe, and 69 (74.1%) patients involved ≥2 lobes (multilobar).

### 3.3. EEG results

Interictal epileptic discharges were observed in all patients during scalp EEG monitoring. There were 33 (35.5%) patients representing unilateral IEDs and 60 (64.5%) patients representing bilateral IEDs. Seizures were recorded in 73 (78.5%) patients, 17 (18.3%) of whom had unilateral epileptic discharges and 56 (60.2%) of whom had bilateral epileptic discharges. Of these 73 patients with recorded seizures, concordance of IEDs and ictal onset rhythms were found in 46 (49.5%) patients.

### 3.4. MEG results

Magnetoencephalography was conducted in 36 (38.7%) patients. The MEG spike sources were observed in 33 (35.5%) patients, among which 23 (24.7%) results were in concordance with the IEDs.

### 3.5. Outcomes of VNS

For all included patients, the median time of the last follow-up was 3.0 (IQR 2.0–4.2) years, ranging from 1.0 to 12.0 years. At the last follow-up, 67 (72.0%) patients were found with reduced seizures, with a median reduction in seizure frequency of 66.7% (IQR 0.0%-100.0%). Of note, 60 (64.5%) patients reported a reduction of ≥50% in seizure frequency, and 15 (16.1%) patients obtained seizure freedom. Seizure outcomes at the last follow-up were assessed using the McHugh and modified Engel seizure outcome classifications (Table 2).

**Table 2:** Seizure outcomes evaluated by modified Engel and McHugh seizure outcome classifications at the last follow-up (≥1 year).

**Class**	**Modified engel description**	**No. of Pts (%)**	**McHugh description**	**No. of Pts (%)**
I	Seizure-free; rare, non-disabling SPS	15 (16.2)	80–100% reduction in seizure frequency	42 (45.1)
II	>90% reduction in seizure frequency; rare CPS	14 (15.0)	50–79% reduction in seizure frequency	18 (19.4)
III	50–90% reduction in seizure frequency	31 (33.3)	<50% reduction in seizure frequency	7 (7.5)
IV	<50% reduction in seizure frequency	33 (35.5)	Magnet benefit only	0 (0.0)
V	/	/	No improvement	26 (28.0)

CPS, complex partial seizure; Pts, patients; SPS, simple partial seizure.

**Figure 3:**
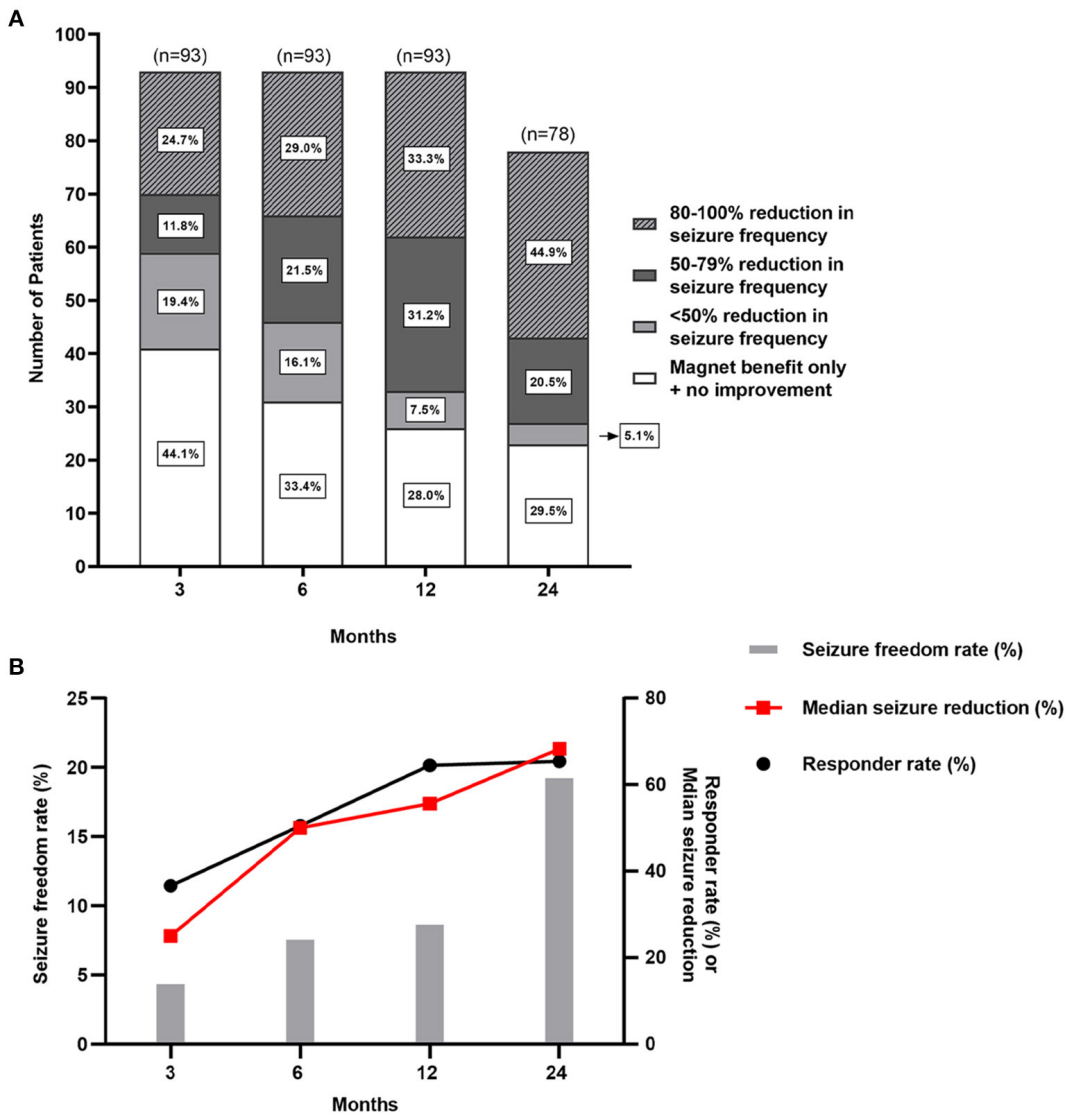
Seizure outcomes of patients with pharmacoresistant epilepsy secondary to encephalomalacia after VNS. **(A)** There are seizure outcomes at 3-, 6-, 12-, and 24-month follow-up after VNS therapy with McHugh outcome classification. **(B)** The responder rate, seizure freedom rate, and median reduction of seizure frequency gradually increase over time.

After VNS therapy, the outcomes of 93 patients with epilepsy secondary to encephalomalacia were shown at the 3-, 6-, and 12-month follow-ups, and the outcomes of 78 patients were shown at the 24-month follow-up (Figure 3). The detailed assessments of VNS outcomes based on the McHugh description at different follow-up time points are shown in Figure 3A. The rates of responder and seizure freedom and the median reduction of seizure frequency were found to gradually increase over time (Figure 3B). At 3, 6, 12, and 24 months of follow-up, the number of responder patients was 34 (36.6%), 47 (50.5%), 60 (64.5%), and 51 (65.4%), respectively; the number of patients with seizure freedom was 4 (4.3%), 7 (7.5%), 8 (8.6%), and 15 (19.2%), respectively; and the median reduction of seizure frequency was 25.0% (IQR 0–77.5%), 50.0% (IQR 0–92.5%), 55.6% (IQR 0–90.9%), and 68.3% (IQR 0–99.9%), respectively.

### 3.6. Analysis of prognostic factors for VNS effectiveness

In the univariate analysis (Table 1), the following factors were found to be associated with VNS effectiveness: the age at seizure onset, duration of epilepsy, the spatial distribution of IEDs, and the encephalomalacia on MRI. The other factors listed in Table 1 were not associated with VNS effectiveness.

**Figure 4:**
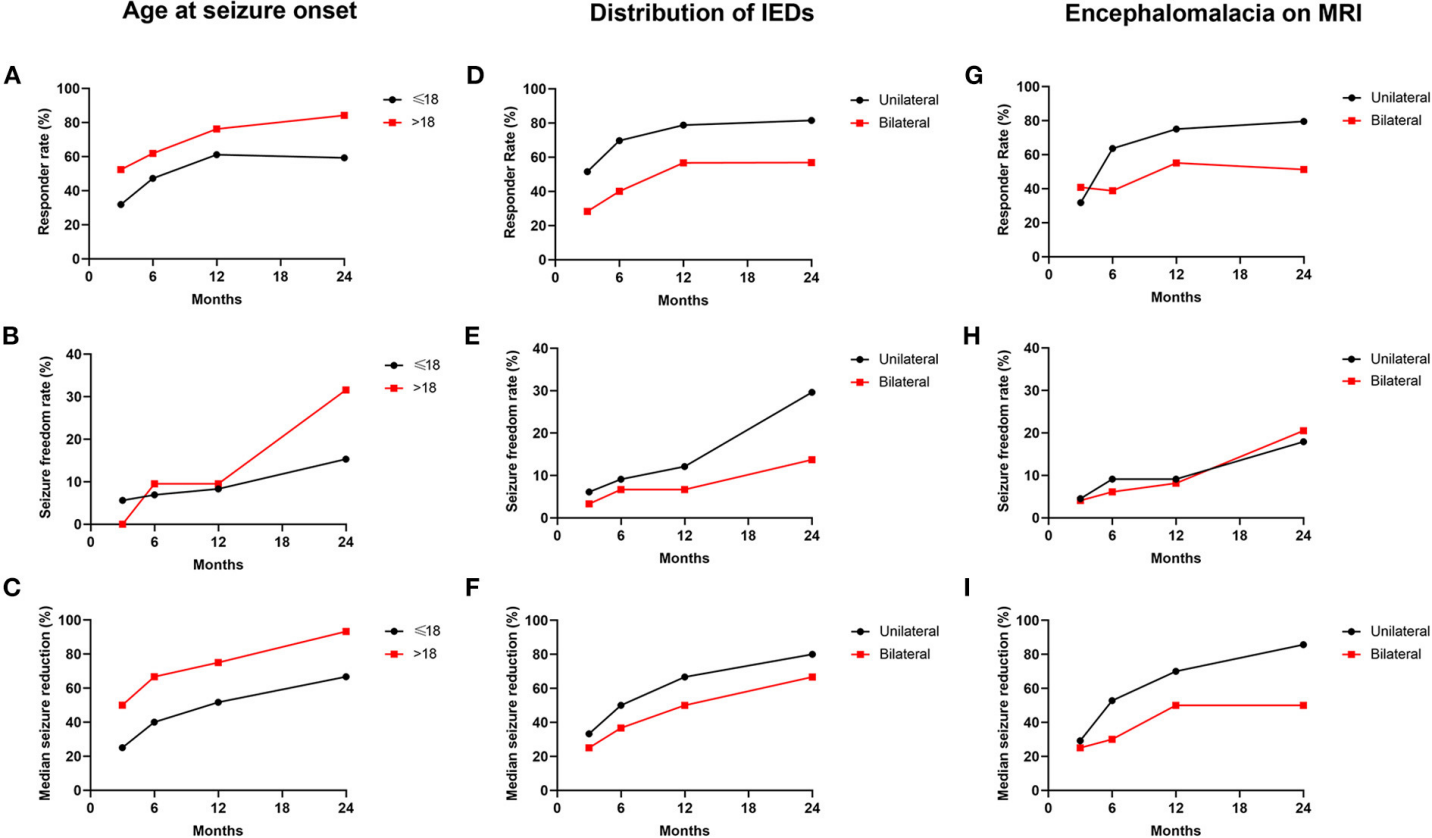
Seizure outcomes in patients classified by predictors of VNS effectiveness. **(A–C)** The responder rate **(A)**, seizure freedom rate **(B)**, and median seizure reduction **(C)** according to the classification of the age at seizure onset. **(D–F)** The responder rate **(D)**, seizure freedom rate **(E)**, and median seizure reduction **(F)** according to the classification of the distribution of IEDs. **(G–I)** The responder rate **(G)**, seizure freedom rate **(H)**, and median seizure reduction **(I)** according to the classification of the distribution of encephalomalacia on MRI.

Variables with statistical significance (*P* < 0.05) in the univariate analysis were then put into the multivariate logistic regression model in a backward manner. After multivariate analysis, the seizure onset in adults (>18 years old) (OR: 0.236, 95% CI: 0.059–0.949) was found to be a positive predictor for VNS effectiveness; the bilateral IEDs (OR: 3.397, 95% CI: 1.148–10.054) and the bilateral encephalomalacia on MRI (OR: 3.193, 95% CI: 1.217–8.381) were found to be negative predictors for VNS effectiveness (Table 3). The responder rate, seizure freedom rate, and median reduction of seizure frequency according to the results of the independent predictors of VNS effectiveness are illustrated in Figure 4.

**Table 3:** Predictors of VNS effectiveness for pharmacoresistant epilepsy secondary to encephalomalacia on multivariate analysis.

**Variables**	**OR**	**95% CI**	***P*-value**
Duration of epilepsy >15 years	2.250	0.757–6.686	0.144
Age at seizure onset >18 years old	0.236	0.059–0.949	0.042^*^
Bilateral IEDs	3.397	1.148–10.054	0.027^*^
Bilateral encephalomalacia on MRI	3.193	1.217–8.381	0.018^*^

CI, confidence interval; IEDs, interictal epileptic discharges; MRI, magnetic resonance imaging; OR, odds ratio; VNS, vagus nerve stimulation;

* *P* < 0.05.

In addition, we also evaluated the prognostic factors for seizure freedom among those patients. After the univariate analysis (Supplementary Table 1), the factor of monthly seizure frequency was found with statistical significance and was then put into the univariate logistic regression model. The monthly seizure frequency (>5) (OR: 3.953, 95% CI: 1.155–13.526) was finally found as a negative predictor for seizure freedom.

## 4. Discussion

Focal encephalomalacia is a common structural brain lesion found in MRI of patients with pharmacoresistant epilepsy. VNS has been used in pharmacoresistant epilepsy for decades. For patients who are unsuitable for resection surgery, VNS may provide better benefits for seizure reduction. However, the long-term seizure outcomes and potential prognostic predictors of VNS in pharmacoresistant epilepsy secondary to encephalomalacia remain unclear. In this study, we first assessed the VNS effectiveness in pharmacoresistant epilepsy secondary to encephalomalacia with a follow-up over 1 year. Out of 93 patients enrolled in this study, 60 (64.5%) patients obtained a reduction of ≥50% in seizure frequency, and seizure freedom occurred in 15 (16.1%) patients. During the follow-up time ranging from 1.0 to 12.0 years, the most frequently reported adverse events included voice hoarse, coughing, and throat pain, while all the side effects above were tolerable and transient. After device implantation, the responder rate, seizure freedom rate, and the median reduction of seizure frequency were all found to gradually increase over time. Those results were consistent with most studies involved VNS effectiveness in pharmacoresistant epilepsy (30, 31), which reported a reduction of or more than 50% in seizure frequency in 45–65% of patients as well as a progressive increase in the overall response to VNS therapy over time. Therefore, VNS therapy was demonstrated to be effective and safe in patients with epilepsy secondary to encephalomalacia. For those who are not suitable for resection surgery, VNS might be a promising therapeutic strategy.

Seizure freedom is generally considered a prominent predictor of life quality in patients with epilepsy. Unfortunately, complete seizure freedom is rarely obtained (6–8%) in patients who underwent VNS surgery (9–12). Among 93 patients with pharmacoresistant epilepsy secondary to encephalomalacia in the present study, 15 (16.1%) patients obtained seizure freedom at the last follow-up, which was higher than that observed in the general population of epilepsy. The relatively high rate of seizure freedom indicated that patients in the small cohort may achieve more improvements in the overall life quality *via* VNS therapy than those with other types of epilepsy. Further studies with larger sample sizes are expected to focus on this problem in the future.

Although the VNS benefit was found more significant in the present cohort than in other types of pharmacoresistant epilepsy, our results confirmed that the complete seizure freedom rate was still less common with VNS than with resective surgery (3). In our previous study focusing on the surgical outcomes in patients with epilepsy secondary to encephalomalacia who received resective epilepsy surgery, ~75.0% of the patients obtained seizure freedom 5 years after surgery (3). A study involving 17 patients with resection of frontal encephalomalacia for pharmacoresistant epilepsy reported that 12 (70%) patients were seizure-free or had only rare seizures after a median of 3 years of follow-up (6). The phenomenon also occurs in other neuromodulation treatments for pharmacoresistant epilepsy (32, 33). Therefore, current neuromodulation techniques are indeed not a substitute for resection therapy for pharmacoresistant epilepsy. However, epilepsy patients with widely distributed encephalomalacia which is involved in eloquent brain regions or bilateral hemispheres are not good candidates for resection. In such conditions, as a palliative treatment, VNS may help reduce the seizure frequency, as well as improve the overall life quality.

In addition to reducing seizure frequency, VNS may also benefit the quality of a patient's life by improving physical disability and neuropsychological disorders (34, 35). Patients with encephalomalacia on MRI usually have various types of initial etiologies, including brain trauma, perinatal hypoxia, meningoencephalitis, previous surgical procedures, and intracranial hematoma, any of which is associated with different degrees of brain damage (6). Therefore, those patients often suffer from neurological and neuropsychological impairments such as physical disability, depression, or anxiety (36, 37). In this study, 31.2% of patients reported a preoperative neurological deficit. Those deficits included hemiparesis, aphasia, ataxia, and persistent vegetative state. Modification of them was also a crucial step during the overall treatment. Multiple preclinical studies on ischemic stroke models have shown that VNS combined with rehabilitation training can significantly improve the recovery of forelimb motor function compared with rehabilitation training without VNS (34). Stimulation of the vagus nerve accelerates the release of neuromodulators, which can promote neuroplasticity throughout the cortex, such as acetylcholine and norepinephrine (38–40). Besides, it is well demonstrated that VNS therapy has benefits on mood, behavior, and cognition for epilepsy patients, independent of reducing seizures (35, 41). Thus, the potential benefits of VNS on psychological and neurological disorders in patients with pharmacoresistant epilepsy secondary to encephalomalacia cannot be ignored. Unfortunately, the neuropsychological disorders and the effectiveness of VNS for those symptoms were not presented in this study, which deserved further exploration in the future.

In the present study, we first evaluated the prognostic predictor of VNS effectiveness in patients with pharmacoresistant epilepsy secondary to encephalomalacia. After multivariate analysis, the age of the seizure onset >18 years was found to predict better effectiveness. Similar results have also been reported in previous studies. In a study recruiting 5,554 epilepsy patients with VNS therapy, the age of epilepsy onset >12 years was found to predict a higher rate of seizure freedom (10). A retrospective analysis of 158 patients with medically pharmacoresistant epilepsy reported that patients with age at seizure onset ≥15 years were ideal candidates for VNS (42). Thus, patients with seizure onset in adults (>18 years old) demonstrated more likely to benefit from VNS therapy than those who had seizure onset in children (≤18 years old). The potential mechanisms of the finding were still unclear. More studies with larger sample sizes are expected to further confirm the phenomenon and explore the underlying mechanisms in the future.

Among the recruited 93 patients, those with unilateral IEDs were found to have a higher rate of responder and seizure freedom, as well as higher median reduction of seizure frequency compared with those with bilateral IEDs at different follow-ups. The important role of EEG features in the prediction of VNS effectiveness for epilepsy has been demonstrated before (43–46). In our previous studies exploring the VNS effectiveness in 42 patients with pharmacoresistant post-encephalitic epilepsy with a follow-up ranging from 1.00 to 11.83 years, patients with focal IEDs were found to have better seizure outcomes than those with generalized IEDs at the last follow-up (18). A study including 144 patients with pharmacoresistant epilepsy reported a significant association between unilateral IEDs and a higher probability of seizure freedom after VNS surgery (44). Notably, it is also the case in resective surgery of patients with epilepsy. In patients with mesial temporal sclerosis who received surgical treatment, bitemporal IEDs indicated bitemporal epileptogenicity and predicted a worse seizure prognosis than unilateral-temporal spike foci (47, 48). The most recognized reason was probably that the bilateral IEDs represented an enlarged epileptogenic zone or a greater epileptogenicity, as the bilateral IEDs were usually accompanied by a bilateral seizure onset zone, a generalized seizure diffusion, and a greater seizure frequency (49, 50). In addition, the bilateral IEDs arising from an interaction of multiple active foci presented a higher degree of epileptogenicity (51). Thus, whether for VNS effectiveness or resection surgery, the spatial distribution of IEDs could be considered a reliable assessment tool for the prognosis of seizure outcome. Besides, the distribution of encephalomalacia foci on MRI was also found associated with VNS effectiveness in the present study. Similar to bilateral IEDs, bilateral encephalomalacia might contribute to the worse seizure outcome *via* similar mechanisms, such as widely distributed brain lesions, generalized seizure propagation, and higher epileptogenicity. In addition, we also evaluated the association between the concordance of the IEDs and ictal onset rhythms or the IEDs and MEG findings with VNS effectiveness in the present study. However, no significant results were obtained after statistical analysis. The results might be biased by the small sample size (36 of 93 patients had MEG results). Further studies with larger sample sizes are expected to focus on this problem in the future.

It was important to acknowledge some limitations of the present study. First, the inherent biases and the relatively small sample size of this single-center retrospective study could not be ignored, and more prospective studies with a larger sample size need to be carried out in the future to make the findings more targeted. Second, some factors that may influence the comprehensive curative effect of VNS in the specific cohort were not included, such as the clinical assessments of neuropsychological problems, behavior disorders, and overall life quality. Third, the 1.5-T MRI equipment used in this study may result in an underestimation of the number of patients with mild encephalomalacia, potentially increasing selection bias. In spite of these limitations, this study suggested the effectiveness of VNS in reducing seizure frequency in patients with pharmacoresistant epilepsy secondary to encephalomalacia. In addition, the age of seizure onset, the spatial distribution of IEDs, and the spatial distribution of encephalomalacia foci on MRI might be independent predictors of VNS effectiveness.

## 5. Conclusion

The present study indicated that VNS therapy was effective in patients with pharmacoresistant epilepsy secondary to encephalomalacia, with an ideal tolerance in patients over a 1-year-follow-up period. Patients with seizure onset in adults (>18 years old), unilateral IEDs, or unilateral encephalomalacia on MRI were found to have better seizure outcomes after VNS therapy.

## Data availability statement

The data analyzed in this study is subject to the following licenses/restrictions: Non-identifying data are available from the corresponding author on reasonable request. Data were not placed in a repository due to the risk of re-identification of participants. Requests to access these datasets should be directed to TL, tianfuli@ccmu.edu.cn.

## Ethics statement

The studies involving human participants were reviewed and approved by the Ethics Committee of Sanbo Brain Hospital, Capital Medical University (SBNK-2017-15-01). Written informed consent to participate in this study was provided by the patients/participants or patients/participants' legal guardian/next of kin.

## Author contributions

TL, GL, and MG contributed to the conceptualization. TL, GL, MG, JW, CT, JD, and JZha contributed to the methodology. MG, ZX, and XK contributed to the formal analysis and investigation. MG contributed to the writing—original draft preparation. TL, GL, MG, JW, CT, JD, JZha, XW, XK, YG, JZha, FZ, and ZX contributed to the writing—review and editing. YG, JZha, and FZ contributed to the investigation. All authors contributed to the manuscript revision, read, and approved the submitted version.
