# Supplementary material for: Vagus nerve stimulation for pharmacoresistant epilepsy secondary to encephalomalacia: A single-center retrospective study
Source: Front Neurol. 2023 Jan 6;13:1074997. doi: 10.3389/fneur.2022.1074997 (PMC9853158; doi:10.3389/fneur.2022.1074997)
Supplement: Supplementary file 1 [file Table_1.DOCX]

Supplementary Material

**Vagus nerve stimulation for** **pharmacoresistant epilepsy secondary to encephalomalacia: a single-center retrospective study**

Mengyi Guo, Jing Wang, Zhonghua Xiong, Jiahui Deng, Jing Zhang, Chongyang Tang, Xiangru Kong, Xiongfei Wang, Yuguang Guan, Jian Zhou, Feng Zhai, Guoming Luan, Tianfu Li

*** Correspondence:**

Guoming Luan, E-mail: luangm@ccmu.edu.cn;

Tianfu Li, E-mail: tianfuli@ccmu.edu.cn.

# 1. Supplementary Table

Supplementary Table 1. Patients’ demographic and clinical features in patients classified by seizure freedom.

| Variable | Total  (n=93) | Seizure freedom (n=15) | Others  (n=78) | *P* Value |
| --- | --- | --- | --- | --- |
| Male, n (%) | 77 (82.8) | 12 (80.0) | 65 (83.3) | 0.501 |
| Age at VNS implantation, year old |  |  |  | 0.323 |
| ≤12 | 20 (21.5) | 2 (13.3) | 18 (23.1) |  |
| >12 | 73 (78.5) | 13 (86.7) | 60 (76.9) |  |
| Age at seizure onset, year old |  |  |  | 0.081 |
| ≤18 | 72 (77.4) | 9 (60.0) | 63 (80.8) |  |
| >18 | 21 (22.6) | 6 (40.0) | 15 (19.2) |  |
| Duration of seizures, year |  |  |  | 0.080 |
| ≤15 | 71 (76.3) | 14 (93.3) | 57 (73.1) |  |
| >15 | 22 (23.7) | 1 (6.7) | 21 (26.9) |  |
| Seizure type, n (%) |  |  |  | 0.563 |
| Focal onset | 82 (88.2) | 13 (86.7) | 69 (88.5) |  |
| Generalized onset | 11 (11.8) | 2 (13.3) | 9 (11.5) |  |
| Monthly seizure frequency |  |  |  | 0.022* |
| ≤5 | 43 (46.2) | 11 (73.3) | 32 (41.0) |  |
| >5 | 50 (53.8) | 4 (26.7) | 46 (59.0) |  |
| Aura, n (%) |  |  |  | 0.336 |
| Yes | 22 (23.7) | 5 (33.3) | 17 (21.8) |  |
| No | 71 (76.3) | 10 (66.7) | 61 (78.2) |  |
| Types of ASMs |  |  |  | 0.232 |
| ≤2 | 62 (66.7) | 12 (80.0) | 50 (64.1) |  |
| >2 | 31 (33.3) | 3 (20.0) | 28 (35.9) |  |
| Etiology |  |  |  | 0.884 |
| Head trauma | 34 (36.6) | 5 (33.3) | 29 (37.2) |  |
| Perinatal hypoxia | 17 (18.3) | 2 (13.3) | 15 (19.2) |  |
| Meningoencephalitis | 17 (18.3) | 4 (26.7) | 13 (16.7) |  |
| Previous surgical procedure | 3 (3.2) | 1 (6.7) | 3 (3.8) |  |
| Intracranial hematoma | 7 (7.5) | 0 (0) | 6 (7.7) |  |
| Unknown | 15 (16.1) | 3 (20.0) | 12 (15.4) |  |
| Age of etiology, year old |  |  |  | 0.186 |
| ≤20 | 61 (65.6) | 7 (46.7) | 54 (69.2) |  |
| >20 | 17 (18.3) | 5 (33.3) | 12 (15.4) |  |
| Unknown | 15 (16.1) | 3 (20.0) | 12 (15.4) |  |
| Interval between etiology and the first seizure, year | | |  | 0.827 |
| ≤8 | 68 (73.1) | 10 (66.7) | 58 (74.4) |  |
| >8 | 10 (10.8) | 2 (13.3) | 8 (10.2) |  |
| Unknown | 15 (16.1) | 3 (20.0) | 12 (15.4) |  |
| Preop neurological deficit, n (%) | 29 (31.2) | 3 (20.0) | 26 (33.3) | 0.376 |
| History of SE, n (%) | 11 (11.8) | 4 (26.7) | 7 (9.0) | 0.074 |
| Spatial distribution of IEDs, n (%) |  |  |  | 0.115 |
| Unilateral | 33 (35.5) | 8 (53.3) | 25 (32.1) |  |
| Bilateral | 60 (64.5) | 7 (46.7) | 53 (67.9) |  |
| Ictal onset rhythms of EEG, n (%) |  |  |  | 0.130 |
| Unilateral | 17 (18.3) | 3 (20.0) | 14 (17.9） |  |
| Bilateral | 56 (60.2) | 6 (40.0) | 50 (64.1) |  |
| Unknown | 20 (21.5) | 6 (40.0) | 14 (18.0) |  |
| Concordance of IEDs and ictal onset rhythms | | |  | 0.148 |
| Yes | 46 (49.5) | 5 (33.3) | 41 (52.6) |  |
| No | 27 (29.0) | 4 (26.7) | 23 (29.5) |  |
| Unknown | 20 (21.5) | 6 (40.0) | 14 (17.9) |  |
| Encephalomalacia on MRI |  |  |  | 0.956 |
| Unilateral | 44 (47.3) | 7 (46.7) | 37 (47.4) |  |
| Bilateral | 49 (52.7) | 8 (53.3) | 41 (52.6) |  |
| Site of encephalomalacia |  |  |  | 0.349 |
| Frontal lobe | 10 (10.8) | 1 (6.7) | 9 (11.5) |  |
| Temporal lobe | 6 (6.5) | 1 (6.7) | 5 (6.4) |  |
| Parietal lobe | 4 (4.3) | 2 (13.3) | 2 (2.6) |  |
| Occipital lobe | 4 (4.3) | 0 (0) | 4 (5.1) |  |
| Multilobar | 69 (74.1) | 11 (73.3) | 58 (74.4) |  |
| Performance of MEG, n (%) |  |  |  | 0.296 |
| Yes | 36 (38.7) | 4 (26.7) | 32 (41.0) |  |
| No | 57 (61.3) | 11 (73.3) | 46 (59.0) |  |
| Concordance of MEG and IEDs |  |  |  | 0.135 |
| Yes | 23 (24.7) | 1 (6.7) | 22 (28.2) |  |
| No | 10 (10.8) | 3 (20.0) | 7 (9.0) |  |
| Unknown^a^ | 60 (64.5) | 11 (73.3) | 49 (62.8) |  |
| The type of stimulator |  |  |  | 0.911 |
| Model 103 | 57 (61.3) | 9 (60.0) | 48 (61.5) |  |
| Model G111 | 36 (38.7) | 6 (40.0) | 30 (38.5) |  |
| Time of the last follow-up, year |  |  |  | 0.065 |
| ≤2 | 28 (30.1) | 2 (13.3) | 26 (33.3) |  |
| 2-6 | 56 (60.2) | 13 (86.7) | 43 (55.2) |  |
| ≤6 | 9 (9.7) | 0 (0) | 9 (11.5) |  |

ASMs, anti-seizure medications; EEG, electroencephalogram; IEDs, interictal epileptiform discharges; MEG, magnetoencephalography; MRI, magnetic resonance imaging; VNS, vagus nerve stimulation; SE, status epilepticus; *, P <0.05; ^a^, MEG was performed in three of these patients, but the spikes sources were not detected.
